# Measurement of patient reported disability using WHODAS 2.0 before and after surgical intervention in Madagascar

**DOI:** 10.1186/s12913-018-3112-z

**Published:** 2018-04-27

**Authors:** Michelle C. White, Kirsten Randall, Dennis Alcorn, Rachel Greenland, Christine Glasgo, Mark G. Shrime

**Affiliations:** 1 Mercy Ships, Department of Medical Capacity Building, Port of Toamasina, Toamasina, Madagascar; 2 Mercy Ships, Department of Medical Capacity Building, Port of Cotonou, Cotonou, Benin; 3 Great Ormond Street Hospital, London, UK; 4 Program in Global Surgery and Social Change, Boston, MA USA; 5 Department of Otolaryngology, Massachusetts Eye and Ear Infirmary, Boston, MA USA

**Keywords:** Global surgery, Surgical outcomes evaluation, Health systems evaluation

## Abstract

**Background:**

Patient reported outcomes (PRO) measure the quality of care from the patient’s perspective. PROs are an important measure of surgical outcome and can be used to calculate health gains after surgical treatment. The World Health Organisation Disability Assessment Schedule (WHODAS) 2.0 is a PRO used to evaluate pre and post-operative disability across a range of surgical specialities. In this study, Mercy Ships, a non-governmental organisation (NGO), used WHODAS 2.0 to evaluate patient reported disability in 401 consecutive patients in Madagascar. We hypothesised that surgical interventions would decrease pre-operative patient reported disability across a range of specialties (maxillofacial, plastic, orthopaedic, general and obstetric fistula surgery).

**Method:**

WHODAS 2.0 was administered preoperatively by face-to-face interview, and at 3 months post-operatively by telephone. Demographic data, American Society of Anesthesiologists (ASA) physical classification score, duration of surgery, length of hospital stay, and in-hospital post-operative complications were collected from a separately maintained patient database. The primary outcome measure was difference in pre- and post-operative WHODAS 2.0 scores.

**Results:**

No differences were seen between the two groups in preoperative disability (*p* = 0.25), ASA score (*p* = 0.46), or duration of surgery (*p* = 0.85). At 3 months 44% (176/401) of patients were available for telephone for postoperative evaluation. All had a significant reduction in their disability score from 8.4% to 1.0% (*p* < 0.001), 17 experienced a post-operative complication, but none had residual disability and there were no deaths. The group lost to follow-up were more likely to be female (65% versus 50%, *p* < 0.05), were younger (mean age 31 versus 35, *p* < 0.05), had longer hospital stays (10 versus 4 days, *p* < 0.001), and were more likely to have experienced post-operative complications (*p* < 0.05).

**Conclusion:**

This study demonstrates that surgical intervention in a LMIC decreases patient reported disability as measured by WHODAS 2.0.

## Background

Patient reported outcomes (PRO) have been defined by the U.S. Food and Drug Administration as “*any report of the status of a patient’s health condition that comes directly from the patient, without interpretation of the patient’s response by a clinician or anyone else*” [1]. In recent years PRO tools have become an increasingly important method of evaluating surgical outcomes. Since 2009, the National Health Service (NHS) in England has collected several PRO measures for four types of surgery: hip replacement, knee replacement, varicose vein and groin hernia surgery, and publishes quarterly reports [2]. Internationally, World Bank has advised countries to ensure that health services are client oriented [3]. Yet, despite this recommendation, PRO’s are still not widely reported in high or low income settings.

New or residual disability after surgery is of concern to both patients and clinicians. Definitions of disability distinguish between the impairment (physical or mental) and the impact the impairment has on the individual’s ability to work, interact socially and to care for themselves [4, 5]. Disability-free survival is said to be an ideal endpoint in surgical outcome studies because it reflects the primary goal for most patients [6], circumvents the problems of using more traditional composite endpoints [7], and can aid shared decision-making [8]. Yet despite this, there remains scant evidence on the impact of disability after surgery. Elderly patients are known to frequently not fully recover after surgery and suffer accelerated disability in the following months and years after surgery, but little is known about the impact of surgical intervention on younger age groups [9–11]. The World Health Organisation Disability Assessment Schedule 2.0 (WHODAS 2.0) is a PRO tool validated in surgical patients [6]. WHODAS 2.0 is publicly available [12] and can be administered quickly by face-to-face or by telephone interview [13].

In low and middle income countries (LMICs), surgery is expensive. 81 million people are estimated to face catastrophic expenditure paying for surgical care [14, 15]. Because of the high cost to patients, it is imperative that surgical care be of high quality and has meaningful impact on patients. Since PRO tools measure quality of care from the patient perspective they have been used to calculate the health gains after surgical treatment in high income countries (HICs) [16] but there is little evidence of use in LMICs.

In LMICs surgical delivery does not only occur in government hospitals. Over 400 surgical non-governmental organizations (NGOs) operate globally [17], and, in some countries, more surgery is performed by NGOs than by the government sector [18]. These surgical NGOs should themselves be held to the same standard, so that the care provided does not merely ‘feel good’ as an act of heroism but provides quality care with meaningful patient impact. In this study, we aim to use a PRO tool to evaluate the patient-centred impact of NGO-delivered surgery. We hypothesized that surgical interventions would decrease patient reported pre-operative disability measured using WHODAS 2.0.

## Methods

The Minister of Health of Madagascar gave permission for the surgical programs including the evaluation and Mercy Ships Institutional Review Board approved the study (MS-2016-003). The requirement for individual written consent was waived.

### Study setting

Mercy Ships is a surgical NGO that delivers free surgeries, training and quality improvement initiatives in coastal sub-Saharan African countries [19]. The ship, the *Africa Mercy,* visits countries at the invitation of the head of State typically spending 10 months in one country before moving to another country. Using 5 operating rooms and 82 beds, a range of surgical services are provided for approximately 1200 patients per field service. Between September 2015 and February 2016, Mercy Ships was based in Madagascar and undertook five specialist surgical programs: general surgery; women’s health; maxillofacial surgery; orthopaedic surgery; and plastic surgery. The different surgical specialities took place in different months during the study period, with only three specialities occurring at any one time.

### Study design and patient population

We aimed to evaluate pre- and postoperative disability in 500 consecutive patients in Madagascar using a prospective observational design. To ensure an even distribution, we aimed for 100 consecutive patients per speciality. Sample size was based on available results from the pilot study [20], which calculated 83 patients per group at a power of 80% and *alpha* of 0.05. All patients gave verbal voluntary consent to participate and the only exclusion criteria was inability to speak French or Malagasy.

### Outcome measure

This study uses the WHODAS 2.0 as a PRO tool to evaluate the impact of surgery on pre- and postoperative disability across a range of surgical specialties.. WHODAS 2.0 has as a 12 or 36 item version. We chose the 12 item version for this study because the 12 item version has been validated in surgical patients [6] and we have previously used the 12 item WHODAS 2.0 as part of a pilot survey reporting patient satisfaction in Madagascar [20]. Additionally the 12 item version is easy to use; can be administered face-to-face or by telephone in about 5 min [13], and is publicly available [12].

WHODAS 2.0 lists 12 activities of daily living and asks individuals to rate the level of difficulty experienced for a task during the previous 30 days [12]. A five-point rating scale (none = 0, mild = 1, moderate = 2, severe = 3, extreme/cannot do = 4) is used; the scores on each activity are combined into a final score out of 60 which is then expressed as a percentage. Higher scores reflect greater disability and a score of 25% or greater is defined as disability [6].

Preoperatively, trained nurses and translators fluent in English, French, and Malagasy, administered the 12-item WHODAS 2.0 (French version) by face-to-face interview. For patients in whom French language was limited, the translators gave additional explanations in Malagasy according to WHODAS administration guidelines. The same nurses and translators telephoned patients 3 months after surgery for post-operative administration of WHODAS 2.0. Up to three separate attempts were made to reach each postoperative patient. Missing data on any survey were handled according to WHODAS manual guidelines [13]: if a single response was missing, the mean value of the remaining responses was assigned; and if more than one response was missing the WHODAS score was not calculated.

Demographic data, American Society of Anesthesiologists (ASA) physical score, duration of surgery, length of hospital stay, and in-hospital post-operative complications were collected from a separately maintained patient database. The ASA score is an international physical status classification system for patients prior to surgery [21]. ASA status is classified as I-VI (I, normal healthy patient; II, patient with mild systemic disease; III, patient with severe systemic disease; IV, patient with severe systemic disease that is a constant threat to life; V, a moribund patient who is not expected to survive without the operation; VI, a declared brain-dead patient whose organs are being removed for donor purposes). Catalogued post-operative complications were unexpected readmission, unplanned return to the OR, unexpected ICU admission, surgical site infection as defined by the US Centres for Disease Control [22], myocardial infarction; stroke, renal failure, gastrointestinal bleed, deep vein thrombosis, pneumonia, sepsis, urinary catheter associated urinary tract infection, central venous catheter associated blood stream infection, coma greater than 24 h, cardiopulmonary arrest, and death.

### Hypothesis and primary outcome measure

The primary hypothesis was that surgical interventions would decrease pre-operative disability. Therefore the primary outcome measure was difference in pre and post-operative WHODAS 2.0 scores.

### Statistical analysis

Statistical analysis was performed with Microsoft Excel Real Statistics. A paired t-test was used for comparison of pre and postoperative WHODAS 2.0. Two sided *p*-values of < 0.05 were considered significant. Mann Whitney test was used to compare non-parametric data (age, sex, ASA score, surgical duration and hospital length of stay) between patients follow-up and those lost to follow-up. Two sided p-values of < 0.05 were considered significant.

## Results

Pre-operative WHODAS 2.0 was recorded in 401 patients. The orthopaedic program only treated 82 patients instead of the 100 predicted, and the plastics program volume was reduced by 60% due to the surgeon suffering a fractured limb and therefore unable to operate. The male: female ratio was 0.42, median age 32 years (Range: 3–73; IQR: 19–46 years); median duration of surgery 115 min (Range: 16–523; IQR: 86–173 min); and median length of hospital stay 7 days (Range: 1–70; IQR: 3–13 days).

**Fig. 1:**
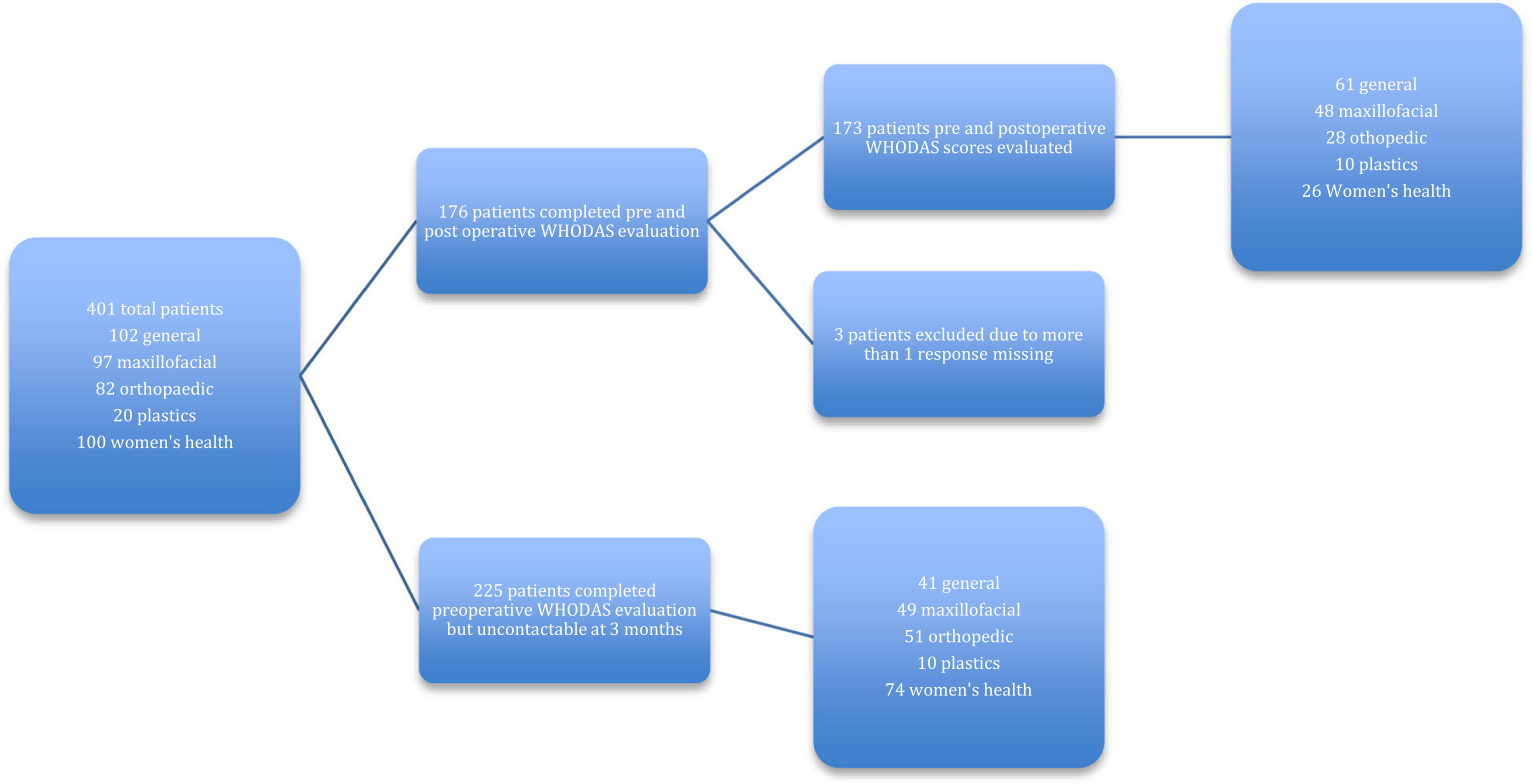
Details of patients with WHODAS 2.0 measured pre- and postoperatively

At 3 months, 176 out of 401 (44%) patients were contacted by telephone for postoperative WHODAS 2.0 evaluation. Three were excluded because more than one WHODAS 2.0 score was missing, leaving 173 for final analysis (Fig. 1).

There was a significant reduction in mean preoperative and postoperative disability scores (8.4% to 1.0%, *p* < 0.001, Table 1). When analysed per surgical speciality, each speciality showed statistically significant reductions in disability.

**Table 1:** Mean (± SD) WHODAS scores by speciality

	Pre-operative (%)	Post-operative (%)	*P* value
Total (*n* = 173)	8.4 ± 0.09	1.0 ± 0.01	< 0.001
General (*n* = 61)	6.6 ± 0.06	1.0 ± 0.02	< 0.001
Maxillofacial (*n* = 48)	9.5 ± 0.08	1.2 ± 0.03	< 0.001
Orthopaedic (*n* = 28)	24.3 ± 0.23	1.9 ± 0.03	< 0.001
Plastics (*n* = 10)	18.8 ± 0.17	3.1 ± 0.05	< 0.001
Women’s Health (*n* = 26)	20.0 ± 0.12	1.0 ± 0.03	< 0.001

Sixty-eight patients out of 401 (17%) met criteria for disability (score > 25%) preoperatively (the number per speciality is shown in Table 2). No patient had residual disability after surgery.

**Table 2:** Number (percentage) of patients per speciality defined as disabled

	Preoperative disability	No preoperative disability
Total (*n* = 401)	68 (17%)	333 (83%)
General (*n* = 102)	2 (2%)	100 (98%)
Maxillofacial (*n* = 97)	6 (6%)	91 (94%)
Orthopaedic (*n* = 82)	23 (28%)	59 (72%)
Plastics (*n* = 20)	6 (30%)	14 (70%)
Women’s Health (*n* = 100)	31 (31%)	69 (69%)

Seventeen out of 401 (4%) patients experienced a post-operative complication and there were no deaths (Table 3). The preoperative disability scores of patients who developed a postoperative complication did not differ from those who did not (*p* = 0.21) although our study was under-powered to detect a difference this small.

**Table 3:** Details of complications

Surgical Specialty	Unexpected ICU admission	Surgical site infection	Unexpected return to the OR	Readmission	Total number of complications
General	0	0	0	0	0
Maxillofacial	1	4	4	3	12
Orthopaedic	0	0	1	1	2
Plastics	0	2	2	0	4
Women’s Health	0	1	4	2	7
Total^a^	1	7	11	6	25

*OR* operating room, *ICU* intensive care unit

^a^Patients experienced more than one complication

Disability scores, demographic and hospital details for patients followed-up at 3 months versus those lost to follow-up are shown in Table 4. The group lost to follow-up were more likely to be female (65% versus 50%, *p* < 0.05), were younger (mean age 31 versus 35, p < 0.05), had longer hospital stays (10 versus 4 days, *p* < 0.001), and were more likely to have experienced post-operative complications (p < 0.05). No differences were seen between the two groups in preoperative disability (*p* = 0.25), ASA score (*p* = 0.46), or duration of surgery (*p* = 0.85).

**Table 4:** Demographic details; duration of surgery, length of hospital stay, and WHODAS 2.0 for patients followed-up and patients lost to follow-up

	Patients followed-up (*n* = 173)	Patients lost to follow up (*n* = 228)	*P* value
Sex (Male: Female ratio)	0.5	0.35	< 0.05
Age ^a^ (years)	35 [20–49]	31 [18–44]	< 0.05
ASA score I:II:III:IV:NR	110: 58: 2: 0: 3	157: 53: 5: 0: 14	0.18
Duration of surgery ^a^ (mins)	114 [85–158]	117 [84–167]	0.46
Length of hospital stay ^a^ (days)	4 [2–10]	10 [4–15]	< 0.001
Preoperative WHODAS 2.0 ^b^	8.36 ± 0.09	13.6 ± 0.13	0.25

*NR* not recorded, *n/s* non-significant

^a^data recorded as median (IQR)

^b^data recorded as mean **± SD**

## Discussion

Across a wide range of surgical specialties, surgical patients treated by an NGO in Madagascar had a significant reduction in self-reported disability as measured using WHODAS 2.0.

It is estimated that globally 143 million additional surgical procedures are needed annually in order to meet the global burden of surgical diseases [23]. In order to ensure quality of surgical scale-up, reliable and easy-to-use measures of surgical quality and patient impact are needed in LMICs. WHODAS 2.0 is a PRO that can measure the patient impact of surgical interventions. This study uses WHODAS 2.0 to demonstrate a reduction in patient-reported disability after NGO surgery in Madagascar.

PRO tools are designed to evaluate what matters to the patient rather than factors clinicians or other stakeholders consider important. Historically clinicians have focused on perioperative mortality rates and incidence of post-operative complications. The 2004 Dindo-Clavien classification [24], grades complications according to patient impact but is not widely used. At the population level, Disability Adjusted Life Years measure the burden of a particular disease and Quality Adjusted Life Years assess the cost-effectiveness of an intervention to treat the disease, but both are focussed from the stakeholder perspective. PRO tools assess what patients think they are able to do and how they feel. Thus PRO tools measure a patient’s health status or health-related quality of life at a single point in time, and are collected through short, self- completed questionnaires. They are widely used in high-income settings [2, 25], but many are difficult to administer and may be difficult for populations with high illiteracy rates to understand. WHODAS 2.0 is simple and quick to use taking approximately 5 min to administer by telephone [13]. Although a wide variety of PRO tools have been documented within National Institute for Health’s Patient Reported Outcomes Measurement Information System, they face two major challenges. Many are not in widespread clinical use, and more work is required regarding quantifying changes for performance and accountability purposes [16]. Our study adds to the evidence that WHODAS 2.0 can be used as a PRO tools to demonstrate patient impact in a LMIC.

Follow up in this study was 44%, which is comparable with other studies investigating surgical outcome in LMICs [26–30]. Most studies evaluate immediate outcome (1–4 weeks), with very few evaluating longer term follow up [28]. Follow-up rates in LMICs are typically lower than those in high income countries (HICs) although even in HICs follow up rates can vary considerably. One HIC study looking at PROs of long-term conditions in primary care reported 38.4% follow up rate [31] while rates of 70% [25] have been reported after hip surgery. Challenges of follow up in LMICs after surgery include: nonfunctioning telephone numbers, transport costs, the requirement for the continued presence of a surgical team to administer post-operative questionnaires and to provide informed responses when difficulties are encountered. In this study, there was no difference between those followed up and those lost to follow up in preoperative disability scores, duration of surgery or length of hospital stay. However, the group lost to follow up was more likely to be female, younger, and to have experienced post-operative complications. Female and younger patients fit the profile of those least likely to have functioning telephone numbers. That they experienced significantly more postoperative complications is difficult to explain. One might consider that in HICs those with complications will likely return to complain or seek further help, but perhaps in a LMIC, they may be unwilling to return for fear of further complications. Since we did not aim to investigate the reasons for lack of follow up we are unable to comment further further.

Post-operative complications were not associated with post-operative WHODAS scores, further illustrating the value of PRO tools in evaluating surgical outcomes and impact. Reliance on more objective and traditional measures of surgical outcome, such as post-operative complications, may be insufficient. In one study in Bangladesh evaluating patient satisfaction in a Government hospital [3], it was noted that patient satisfaction was unrelated to surgical outcome, and that respect and politeness of providers, as opposed to their technical ability, was the greatest predictor of patient satisfaction. Similar findings have been seen in cleft lip and palate surgery in Nigeria [30]. Patients satisfied with the service and care they received are more likely to be committed to future treatments and would recommend the service to others [30, 32]. Also, since satisfaction is heavily influenced by things like the cost of surgery and the attitude of providers [30], traditional measurements of mortality and morbidity are insufficient to monitor patient satisfaction and impact. This study shows how WHODAS as a PRO can show a positive patient impact (significant reduction in disability) after surgery in a LMIC.

This study has several limitations. Working cross culturally as well as in different languages represents a challenge to data collection and whilst we ensured that we used experienced translators, information can be lost in translation. Baseline data was obtained through face-to-face interview whereas follow-up data by telephone and while WHODAS 2.0 is designed to be administered in both ways, we are unable to rule out any effect from the mode of interview. Our study presents paired data but did not adjust for confounders, and was underpowered to accurately compare the postoperative disability scores of those who experienced postoperative complications with those who did not. Since the group lost to follow up had more females, younger age, longer length of stay and more complications, the results may not be generalizable to the entire Mercy Ships surgical population. Bias may also be introduced by those lost to follow up in that they may have not wanted to share negative results. Mercy Ships provides surgery for free, and this may also affect patient’s willingness to report a negative outcome. Despite these limitations, our study has a number of strengths. The study demonstrates the use of WHODAS as a PRO tool in LMICs to show a reduction in disability across a wide range of specialties; the follow up rate of 44% is relatively high and the numbers are large compared to other follow up studies in LMICs.

## Conclusion

Our study shows that there was significant reduction in patient reported disability after surgical intervention in a LMIC. We recommend that WHODAS 2.0 is used as a PRO tool by NGOs and other stakeholders involved in the delivery of surgical care to monitor patient-centered impact of surgical interventions. This will ensure that surgical health services remain patient oriented as recommended by World Bank [3].

## Abbreviations

ASA: American society of anesthesiology
LMIC: Low and middle income countries
NGO: Non-Governmental organization
PRO: Patient reported outcomes
WHODAS: World Health Organisation Disability Schedule
